# Structural and functional insights into the LBD family involved in abiotic stress and flavonoid synthases in *Camellia sinensis*

**DOI:** 10.1038/s41598-019-52027-6

**Published:** 2019-10-30

**Authors:** Xueying Zhang, Yuqing He, Wenda He, Hui Su, Yuefei Wang, Gaojie Hong, Ping Xu

**Affiliations:** 1 Department of Tea Science, Zhejiang University, Hangzhou, 310058 China; 2 State Key Laboratory for Managing Biotic and Chemical Threats to the Quality and Safety of Agro-products, Institute of Virology and Biotechnology, Zhejiang Academy of Agricultural Sciences, 198 Shiqiao Road, Hangzhou, 310021 China

**Keywords:** Plant stress responses, Plant stress responses, Secondary metabolism, Secondary metabolism

## Abstract

Lateral organ boundaries domain (LBD) proteins are plant-specific transcription factors that play a crucial role in growth and development, as well as metabolic processes. However, knowledge of the function of LBD proteins in *Camellia sinensis* is limited, and no systematic investigations of the LBD family have been reported. In this study, we identified 54 LBD genes in *Camellia sinensis*. The expression patterns of *CsLBDs* in different tissues and their transcription responses to exogenous hormones and abiotic stress were determined by RNA-seq, which showed that CsLBDs may have diverse functions. Analysis of the structural gene promoters revealed that the promoters of *CsC4H*, *CsDFR* and *CsUGT84A*, the structural genes involved in flavonoid biosynthesis, contained LBD recognition binding sites. The integrative analysis of *CsLBD* expression levels and metabolite accumulation also suggested that CsLBDs are involved in the regulation of flavonoid synthesis. Among them, CsLOB_3, CsLBD36_2 and CsLBD41_2, localized in the nucleus, were selected for functional characterization. Yeast two-hybrid assays revealed that CsLBD36_2 and CsLBD41_2 have self-activation activities, and CsLOB_3 and CsLBD36_2 can directly bind to the *cis*-element and significantly increase the activity of the *CsC4H*, *CsDFR* and *CsUGT84A* promoter. Our results present a comprehensive characterization of the 54 *CsLBDs* in *Camellia sinensis* and provide new insight into the important role that *CsLBDs* play in abiotic and flavonoid biosynthesis.

## Introduction

Plants have evolved a variety of biochemical and physiological mechanisms to survive under temporary or continuous environmental challenges^1,2^. Transcription factor (TF) families play important roles in plant growth, development and environmental stress responses^3,4^. As plant specific TFs, LATERAL ORGAN BOUNDARIES (LBD) genes can be identified by a highly conserved LBD domain, which acts in the boundary of plant organs to regulate the development of leaves, inflorescences, roots and microspores^5,6^. LBD genes also play important roles in metabolic processes in higher plants, such as anthocyanin and nitrogen metabolism^7^.

As the complete reference genomes of more species are sequenced, the LBD gene family has been identified in several plants. In *Arabidopsis* and *zea mays*, 43 and 44 LBD members have been found, respectively, 35 have been identified in rice, 57 in poplar, 58 in *malus domestica*, 28 in *brachypodium* and 46 in tomato^6,8–13^. Generally, LBD proteins are defined by an N-terminal LBD domain. The characteristic LBD domain comprises a C-domain containing four conserved cysteines with spacing (CX2CX6CX3C) required for DNA binding activity. Moreover, the LBD domain contains a Gly-Ala-Ser (GAS) block and a leucine zipper-like coiled-coil motif (LX6LX3LX6L), which includes five hydrophobic amino acids separated by six variable amino acid residues responsible for protein dimerization^14^. According to characteristic sequence motifs, LBD genes are divided into two classes. The majority of LBD genes belong to Class I, as they contain a perfectly conserved CX2CX6CX3C zinc finger-like domain and an LX6LX3LX6L leucine zipper-like coiled-coil motif. Usually, members of the Class I group are involved in plant development and auxin signal transduction cascades^14–16^. In contrast, Class II LBD genes, which possess a conserved zinc finger-like domain cannot form coiled-coil structures^8,14^.

In *Arabidopsis*, several LBDs have been characterized. For example, *AtASL4* (AtLOB) is predominantly expressed in the proximal base of lateral tissues and interacts with various transcription factors and proteins to participate in early leaf development^5^. The genes *AtLBD17*, *AtLBD18* and *AtLBD29* can regulate the development of lateral roots and callus formation, and can establish a molecular link between auxin signaling and the plant regeneration program^17–19^. In Class II, *AtAS2* (*AtLBD6*) not only participates in leaf near-paraxial polarity, but also plays a role in floral development by synergistically regulating the differentiation of border cells in flower organs through AS1 and JAG^20^. *AtLBD38* and *AtLBD39* play a role in nitrogen metabolism and anthocyanin synthesis^7,21^. *AtLBD20* is a root specific LBD gene that negatively regulates the responses to fungal infection^22^. The functions of LBD genes have also been studied in other species. For example, *OsIG1* (homologous to *AtAS2*) can affect leaf lateral growth by regulating the division and differentiation of vesicular cells between vascular bundles^23^. *OsLBD37* and *OsLBD38* are involved in the regulation of rice plant heading date and crop yield^24^. *ZmIG1* plays a key role in the regulation of female gamete development and leaf axial differentiation^25^. LBD1 and LBD4 in poplars act together on the secondary phloem, while *PtaLBD15* and *PtaLBD18* are specifically expressed in the secondary xylem, indicating that the LBD family is involved in secondary growth during xylem formation^10^.

Tea is the world’s most popular beverage and offers a wealth of health benefits. Previous studies have documented that flavonoids have strong antioxidant activity as well as many other medicinal properties that act against a variety of human diseases^26^. Catechins are a major component of flavonoids and are synthesized through the flavonoid pathway, which has been intensively investigated in several plant species^27–30^. Firstly, conversion of phenylalanine to chalcon, a common precursor in the flavonoid biosynthetic pathway, is catalyzed by phenylalanine ammonia-lyse (PAL), cinnamate 4-hydroxylase (C4H), 4-coumarate-CoA ligase (4CL) and chalcone synthase (CHS)^31,32^. Subsequently, chalcon is converted into to leucoanthocyanidin under the catalyzation of chalcone isomerase (CHI), flavanone 3-hydroxylase (F3H), flavonoid 3′-hydroxylase (F3′H), flavonoid 3′5′-hydroxylase (F3′5′H), and dihydroflavonol 4-reductase (DFR)^33,34^. Then, leucoanthocyanidins are either catalyzed by leucoanthocyanidin 4-reductase (LAR) to produce Catechins (C) and gallocatechin (GC), or by the sequential action of anthocyanidin synthase (ANS) and anthocyanidin reductase (ANR) to form epicatechin (EC) and epigallocatechin (EGC), respectively^35^. Proanthocyanidins (PAs, also called condensed tannins) are oligomers and polymers of non-galloylated catechins (C, GC, EC and EGC), which can be catalyzed by galloyl-1-*O*-β-D-glucosyltransferase (UGGT) and epicatechin: 1-*O*-galloyl-β-D-glucose *O*-galloyltransferase (ECGT) to synthesis galloylated catechins (catechin gallate, epicatechin gallate, epigallocatechin gallate and gallocatechin gallate)^36^.

Catechin content is greatest in new tea shoots, and gradually decreases with the maturation of tissues and the growth of plants^37^. LBD genes play essential roles in plant growth and development and are involved in anthocyanin synthesis and nitrate metabolism^7,21^. However, the function of LBD genes in *Camellia sinensis* remains largely unexplored. Completion of the genome-sequencing project for *Camellia sinensis* has made it possible to identify LBD genes on a genome-wide scale^38,39^. We identified 54 LBD genes in the *Camellia sinensis* genome through database searches, and classified them according to their homology with LBD genes in *Arabidopsis*. We analyzed their sequence phylogeny, genomic structure, conserved domains and evolutionary mechanisms. We also investigated *CsLBD* gene expression patterns in different tissues and in response to MeJA and other different abiotic stresses. Furthermore, we characterized the function of selected LBD genes by subcellular localization, transactivation analysis, yeast one-hybrid assay and dual-luciferase assay to demonstrate that three LBD members have different effects on flavonoid synthesis. Our findings will serve as a foundation for further research into the roles of CsLBDs in flavonoid synthesis.

## Results

### Identification and annotation of LBD genes in tea plant

To identify the LBD gene family members in tea plant, 78 LBD protein sequences, 43 from *Arabidopsis* and 35 from *O. sativa*, were chosen to screen the tea genome database (*Camellia sinensis* var. *sinensis*). FGENESH (http://www.softberry.com/berry.phtml) was applied to confirm these predicated LBD sequences and the ExPASy proteomics server (http://www.expasy.ch/prosite/) was used to check the domains of the LBD sequences. A total of 54 LBD genes were identified in *Camellia sinensis* var. *sinensis*. These LBD genes were predicted to encode proteins 123–401 amino acids in length, with putative molecular weights (MWs) ranging from 13.39 to 45.04 and isoelectric points (pI) ranging from 4.7 to 9.37 (Supplementary Dataset 1). To avoid confusion, the 54 CsLBDs were named according to their homology with *Arabidopsis* LBDs. However, not every LBD member of *Camellia sinensis* corresponded to a gene in *Arabidopsis*. Genes with the same homology were further distinguished by an extra number.

### Sequence alignment and phylogenetic analysis of LBD genes

Multiple sequence alignment of LBDs in *Camellia sinensis* revealed the presence of the CX2CX6CX3 zinc finger-like domain signature in the N terminus of all genes with the exception of CsLBD29. This domain, is required for DNA-binding activityGly-Ala-Ser (GAS) block and leucine zipper-like coiled-coil (LX6LX3LX6L) motifs were located at the C terminus, and are responsible for protein dimerization. In the GAS block, CsLBD23 and CsLBD24 had no corresponding sequences, whereas all CsLBD proteins contained conserved residues in the (D/N) PX2G motif. As in other species, the leucine zipper-like motif (LX6LX3LX6L) was only observed in CsLBD class I proteins, suggesting that the classes might have distinct functions (Supplementary Fig. S1).

**Figure 1:**
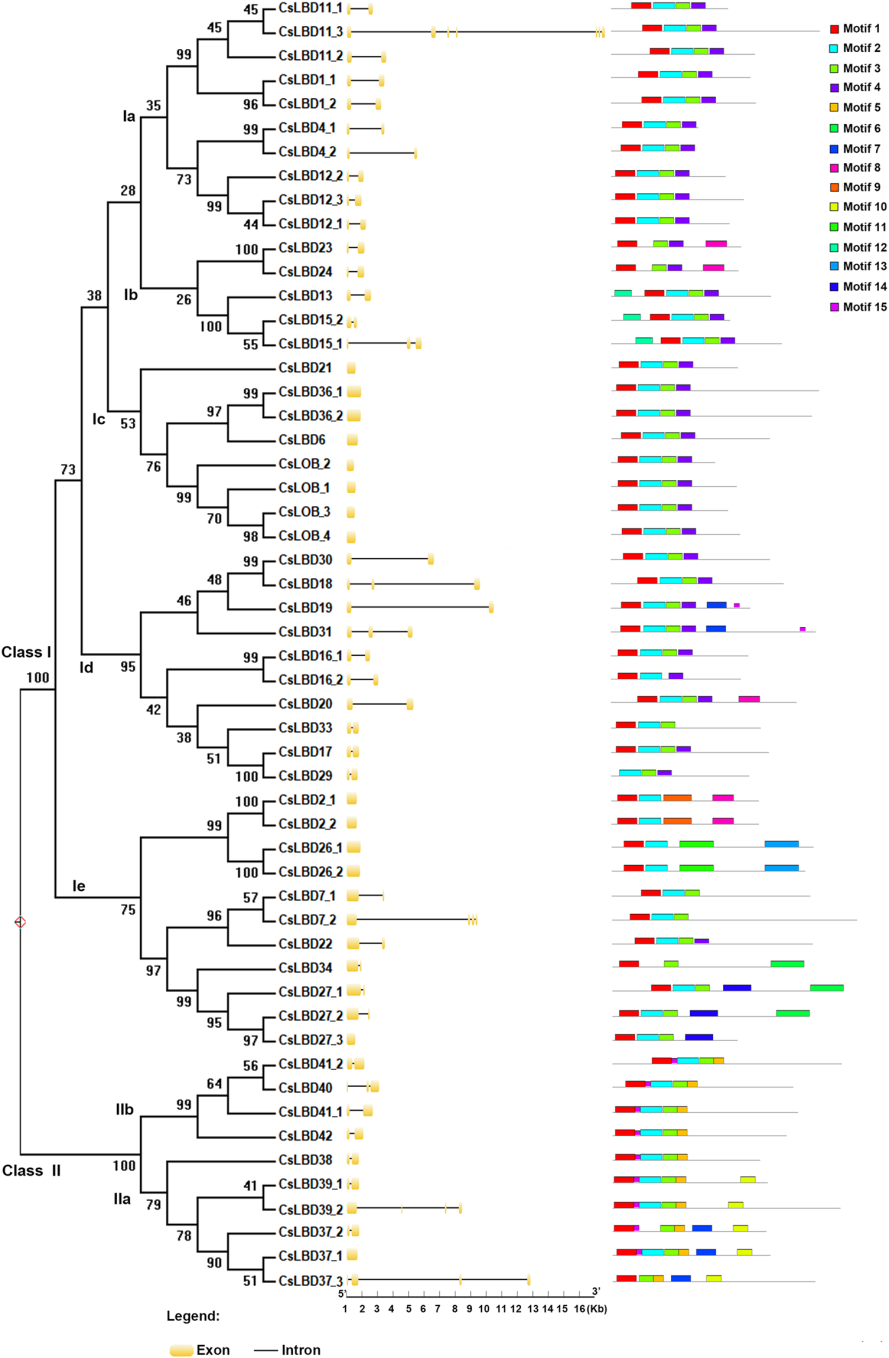
Phylogenetic analysis, and identification of intron-exon and conserved motifs in 54 LBDs in *Camellia sinensis*. A phylogenetic tree of 54 CsLBDs was constructed using MEGA X by the NJ method with 1000 bootstrap replicates. Introns and exons are represented by a black line and orange box, respectively. Conserved motifs are indicated by a colored box numbered from 1 to 15.

Exon/intron analysis showed that the number of *CsLBD* exons ranged from one to seven. The majority of the *CsLBD* genes contained two exons (31 genes), 17 had no introns, 4 had two introns, *CsLBD37_3* and *CsLBD7_2* had four introns, *CsLBD39_2* had five and *CsLBD11_3* had seven. The size of LBD gene loci ranged from 459 (*CsLBD_2*) to 16697 (*CsLBD11_3*) nucleotides (Fig. 1). Most of the LBD exon/intron structures were clustered together in the phylogenetic tree, indicating evolutionary conservation of the gene structure.

MEME online software was used to predict the motifs in CsLBD protein sequences, and fifteen motifs were indicated in the LBD protein structure (Fig. 1). Nearly all members of the CsLBD family contained motifs 1, 2 and 3, suggesting that these motifs are essential for the functions of the LBD gene family. Motif 5 was only present in Class II. LBD proteins were clustered into subgroups based on their motif compositions and exon/intron structures.

**Figure 2:**
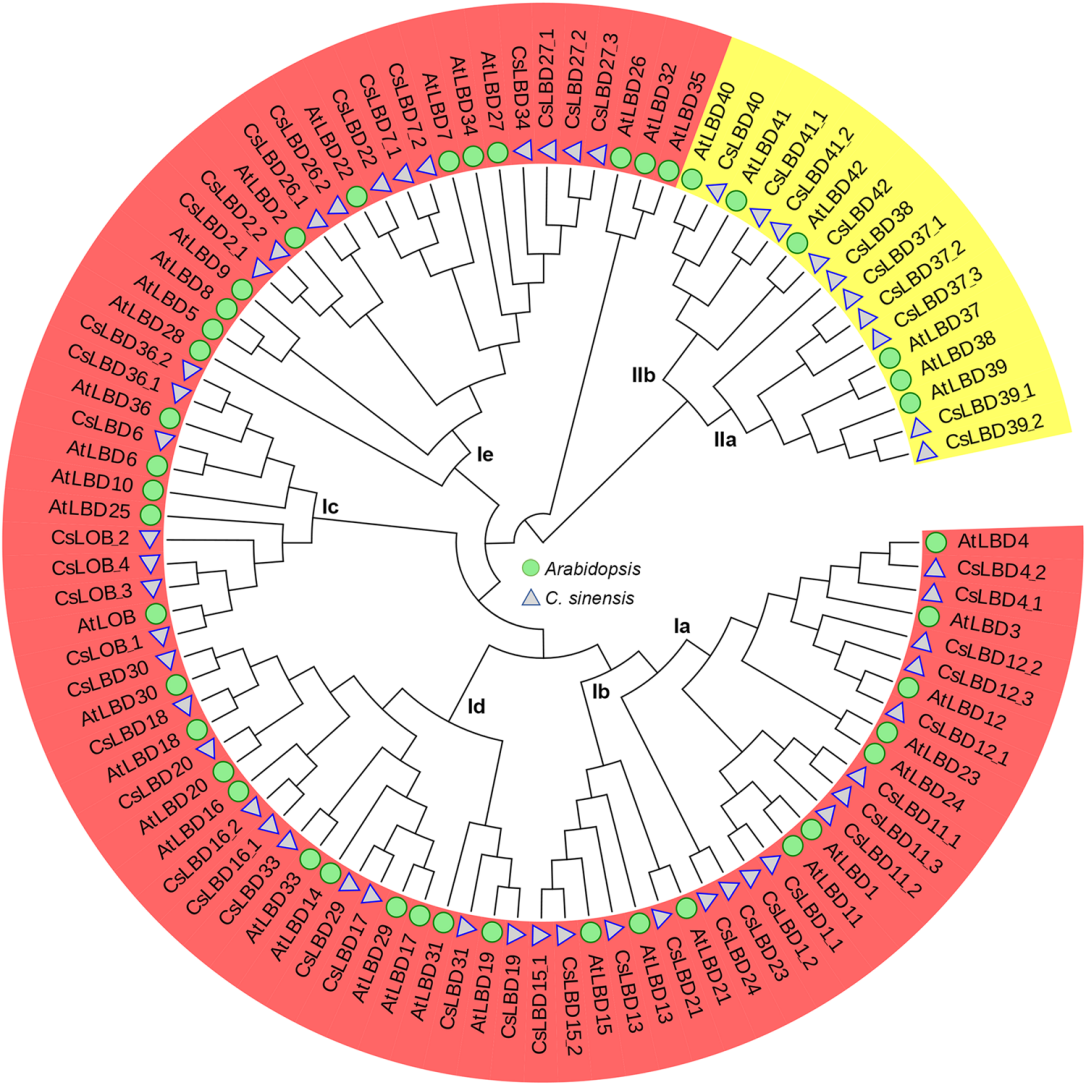
Phylogenetic tree of LBDs from *Camellia sinensi*s and *Arabidopsis*. Amino acid sequences were aligned using Clustal W, and MEGA X software was used to construct the phylogenetic tree by the NJ method with 1000 bootstrap replicates. All LBD proteins were divided into Class I and Class II and then divided into five (Ia, Ib, Ic, Id and Ie) and two (IIa and IIb) subgroups, respectively.

A phylogenetic tree was constructed with the full length protein sequences to examine the evolutionary patterns of 54 LBDs in *Camellia sinensis* and 43 in *Arabidopsis* using the MEGA X program. As shown in Fig. 2, all LBD proteins were divided into two classes, named Class I and Class II. These were further divided into five (Ia, Ib, Ic, Id and Ie) and two (IIa and IIb) subgroups, respectively. Class I comprised 44 CsLBDs and 36 AtLBDs, while Class II comprised 10 CsLBDs and 6 AtLBDs (Fig. 2).

### *CsLBD* expression patterns of in *Camellia sinensis*

**Figure 3:**
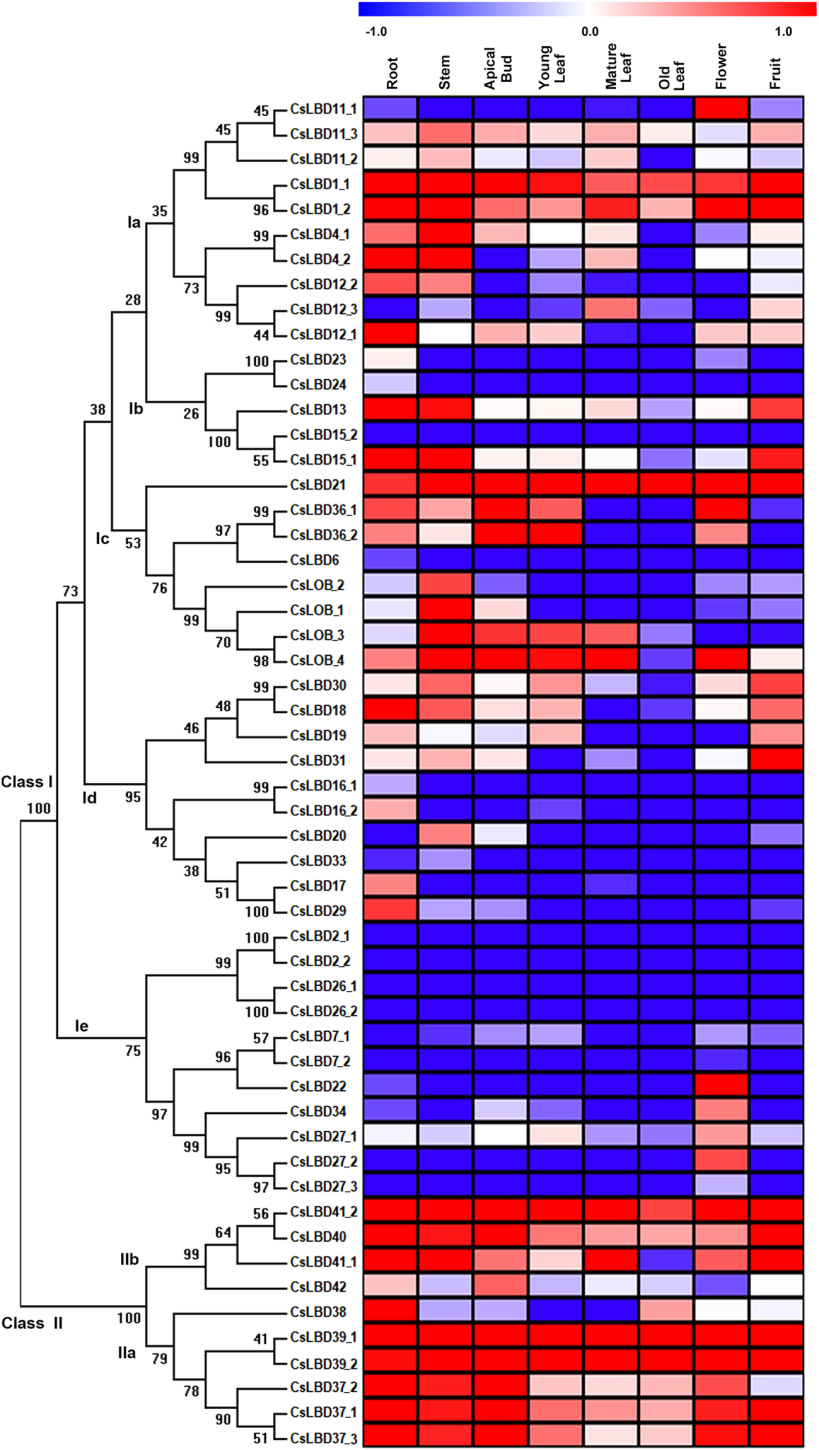
Expression patterns of *CsLBDs* in different tissues in *Camellia sinensis*. The expression levels of *CsLBD* genes in eight tissues (Root, Stem, Old leaf: germinated in previous years, Mature leaf: geminate in the spring and are harvested in the autum, Young leaf: the first and second leaf follows the apical bud, Apical bud: unopened leaves on the top of activity growing shoots, Flower and Fruit) of tea plant were calculated using Log_10_(FPKM).

**Figure 4:**
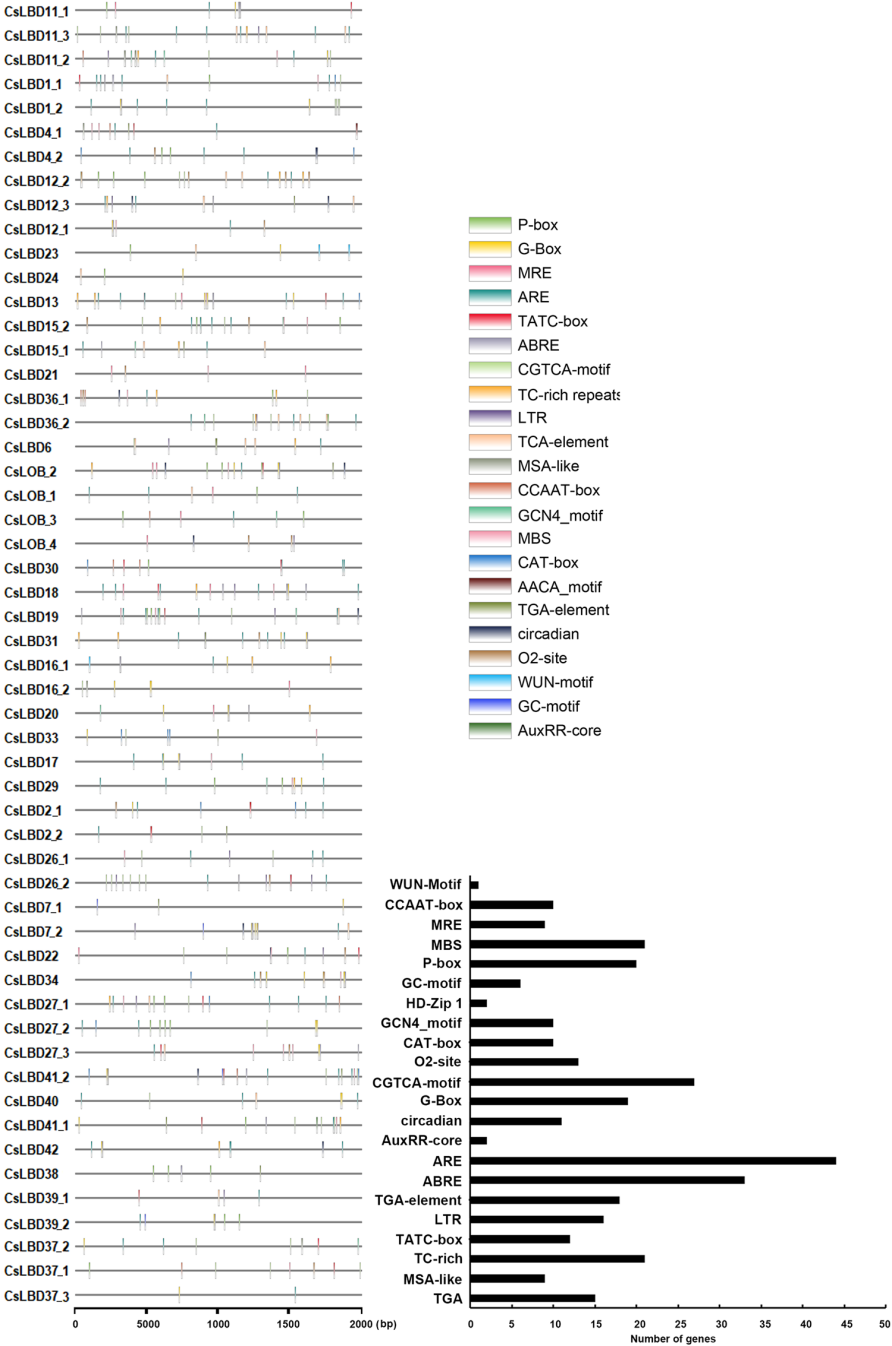
*Cis*-element analysis of the *CsLBD* promoters. Plant CARE was used to identify the putative *cis*-acting element distribution in 2000 bp promoter sequences of 54 *CsLBDs*.

To explore the organ-specificity of LBD family members, we examined the abundance of 54 *CsLBD* transcripts in eight representative tissues of *Camellia sinensis* cv. Shuchazao, comprising apical bud, young leaf, mature leaf, flower, fruit, old leaf, stem and root tissue (Fig. 3). The expression of six members in subclass Ia was detected at least in one of the organs. *CsLBD1_1* and *CsLBD1_2* shared similar transcript profiles and were mainly expressed in fruit; *CsLBD4_1* and *CsLBD4_2* were mainly expressed in stems; and *CsLBD11_1* and *CsLBD12_1* were predominantly expressed in flower and root tissues, respectively. Only two members of subclass Ib (*CsLBD13* and *CsLBD15_1*) showed high expression levels in root. All members of subclass Ic were detected in at least one organ, with the exception of *CsLOB_2* and *CsLBD6*. Transcripts of *CsLBD21* were detected in all organs; CsLBD36_1 was mainly expressed in apical bud and flower tissue, while *CsLBD36_2* accumulated in apical bud and young leaf tissue; and *CsLBD_1* and *CsLBD_4* shared similar expression patterns and were predominantly expressed in root tissue. Members of subclasses Id and Ie showed very low or undetectable expression levels in all tested tissues, excepted *CsLBD18*, *CsLBD31* and *CsLBD22*. *CsLBD18* was mainly expressed in roots, *CsLBD31* in fruit and *CsLBD22* in flowers. All LBDs in Class II except *CsLBD42* showed high expression in most organs. Among them, transcripts of *CsLBD39_1*, *CsLBD39_2* and *CsLBD41_2* were detected in the whole plant, whereas CsLBD37_1/2/3, *CsLBD38*, *CsLBD40* and *CsLBD41_1* were only highly expressed in roots. Furthermore, we examined the transcript abundance of 10 selected *CsLBDs* in different tissues of tea plant cultivar (Longjing 43) tissues using quantitative RT-PCR (qRT-PCR) (Supplementary Fig. S2). The expression patterns of nine genes were highly consistent with their transcriptomic profile from RNA-seq and their Pearson’s correlation coefficient R = 0.84. Both q-RT-PCR and RNA-seq data indicated that the no *CsLBD26_2* transcripts were present levels in tested tissues. Previous studies have indicated that *cis*-acting elements present in gene promoter regions are closely related to their own expression pattern^40,41^. A number of common *cis*-elements were identified, most of which were involved in plant growth and development. CAT-box and GCN4_motifs were identified in ten *CsLBD* promoters, and are related to meristem and endosperm expression respectively. Both *CsLOB_3* and *CsLBD36_2* possessed with GCN4_motif and highly expressed in apical bud and young leaf. Six promoters contained MSA-Like elements, which are involved in cell cycle regulation (Fig. 4, Supplementary Dataset 2). High levels *CsLBD21* and *CsLBD41_2* transcripts were detected throughout all tissues, and their promoters contained growth-related *cis*-elements (circadian, CAT-box and MSA-like). This suggests that CsLBDs play important roles in biological processes, as well as regulating plant growth and development.

### Expression profiles of *CsLBDs* in response to abiotic stress

**Figure 5:**
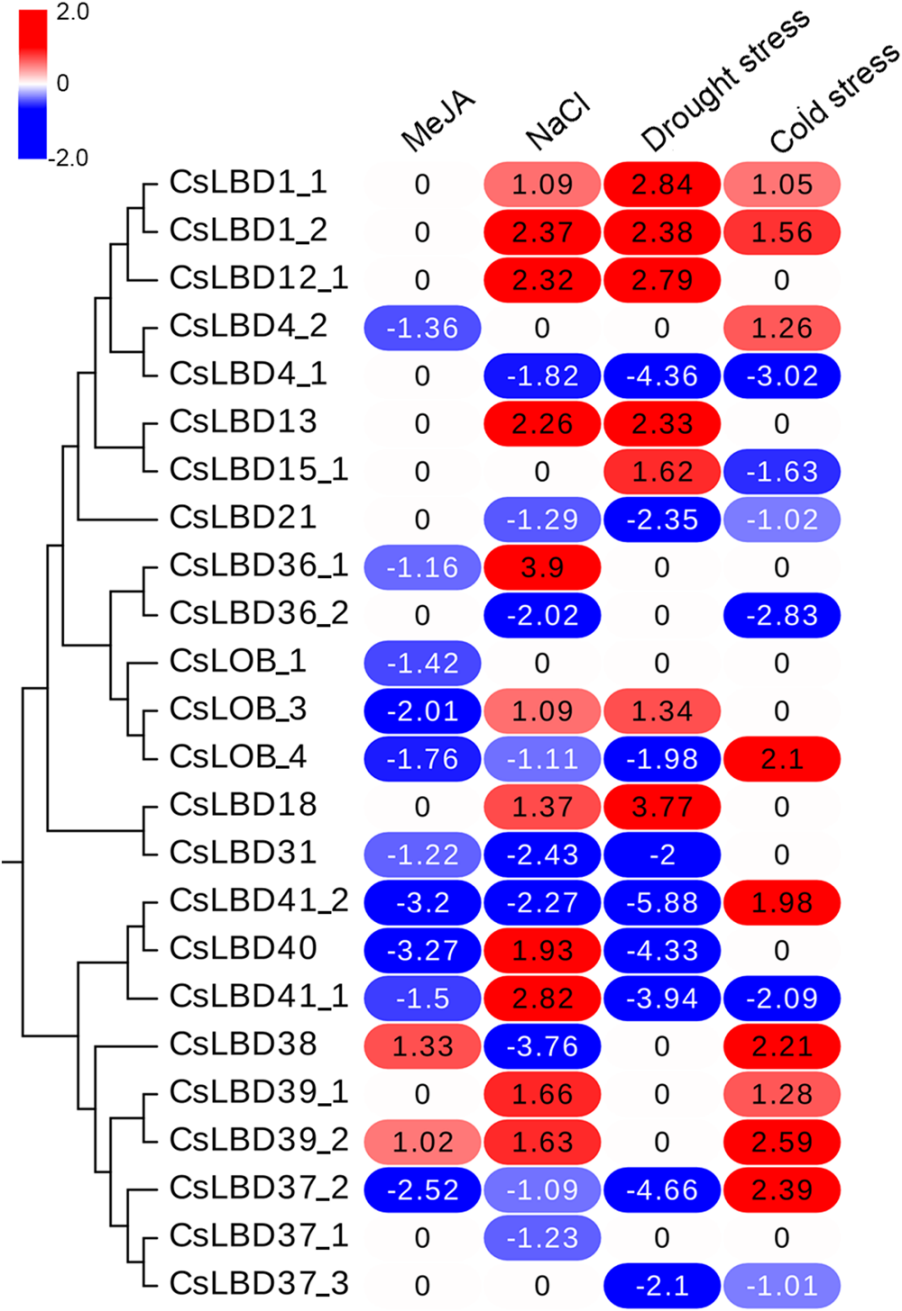
Expression pattern of *CsLBDs* in response to MeJA and abiotic stress. Genevestigator analysis of *CsLBDs* using the Log_2_(Experiment/Control) in response to MeJA, NaCl, drought and low temperature.

Many *CsLBD* promoters included *cis*-elements responsible for stress responses, including 16 low-temperature responsive elements (LTR-element), 21 defense and stress response elements (TC-rich repeats), and nine MYB binding sites related to drought-inducibility (Fig. 4, Supplementary Dataset 2). To investigate the potential functions of *CsLBD* genes in response to MeJA and abiotic stresses, we searched the published literature for relevant microarray data. Expression patterns of the *CsLBD* genes in response to MeJA and abiotic stress (Cold stress, NaCl stress and drought stress) are shown in Fig. 5. Among the 54 predicted genes, 24 gene expression profiles were obtained in the Genevestigator analysis. It is possible that either the transcript abundance of the 30 genes was too low to be detected or there were no changes following treatments. Following MeJA treatment, the expression of most *CsLBDs* was unchanged or repressed; only *CsLBD38* and *CsLBD39_2* were induced significantly. Among 24 *CsLBDs*, 17 showed similar responses to three abiotic stresses: ten were induced, six LBDs were suppressed, and *CsLOB_1* was unchanged. *CsLBD13* was induced by drought and repressed by cold stress, *CsLBD_4*, *CsLBD41_2*, *CsLBD38* and *CsLBD39_2* were repressed by both NaCl and drought stress but induced by cold stress. *CsLBD40* and *CsLBD41_1* were induced by NaCl stress and suppressed by drought and cold stress.

### Integrative analysis of *CsLBD* expression levels and metabolite accumulation in tea tissues

**Figure 6:**
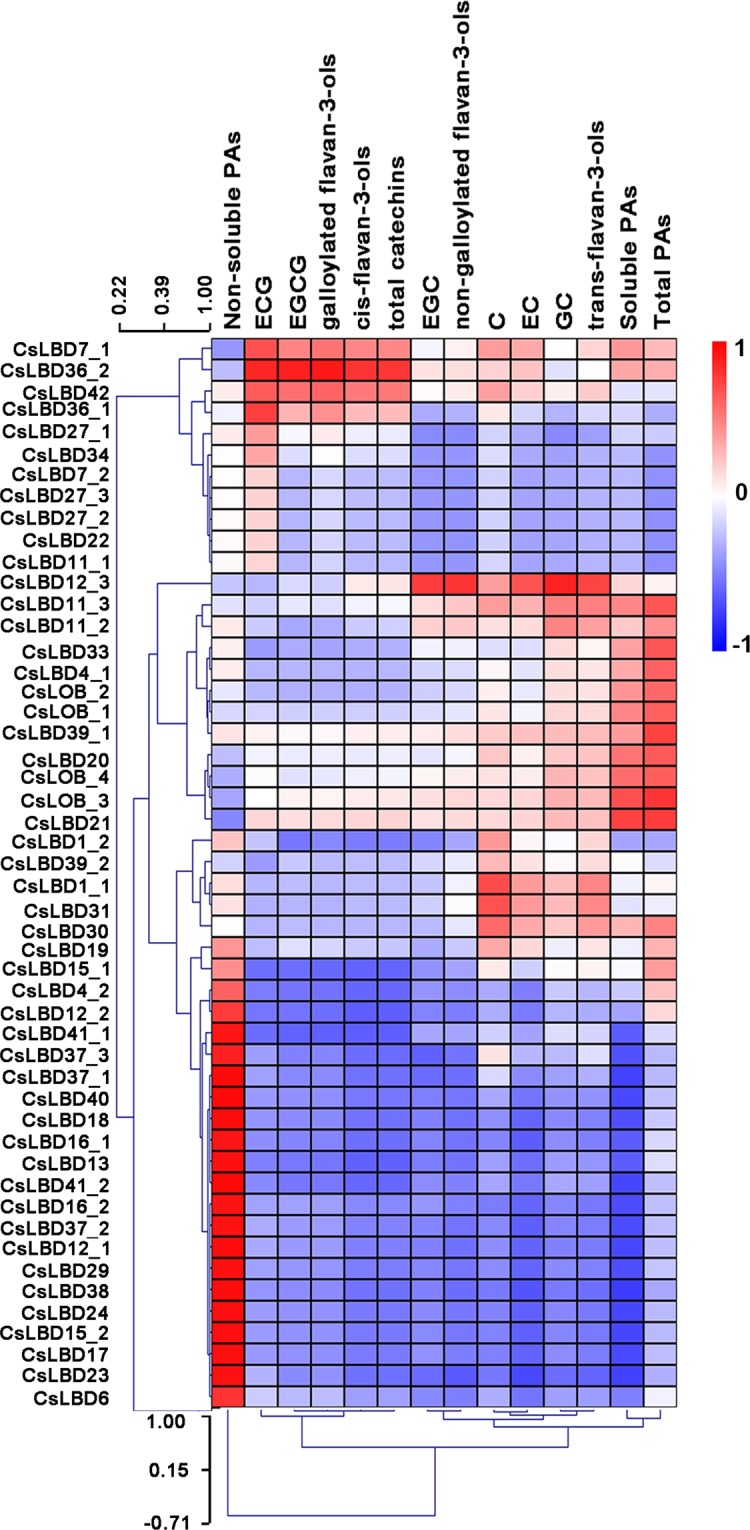
Integrative analysis of *CsLBD* expression levels and metabolite accumulation in tea tissues. Correlation analysis with 13 metabolites and 50 transcripts of *CsLBD*. R > 0.5: Positive correlations; R < −0.5: negative correlation.

Transcriptomic profiles and metabolite activities of eight representative tissues of tea plant was used to establish gene to metabolite networks. Among 54 *CsLBDs*, no expression of *CsLBD2_1*, *CsLBD2_2*, *CsLBD26_1* and *CsLBD26_2* was detected in any of the tested tissues. We preformed correlation analysis of 13 flavonoid metabolites and another 50 LBD transcripts and identified 47 positive correlations (R > 0.5) and 126 negative correlations (R < −0.5) (Fig. 6). There were 19 *CsLBDs* that had clear negative correlations with total catechins, of which four members belonged to subclass Ia, five to subclass Ib, four to subclass Id, three to subclass IIa and three to subclass IIb. Particularly interesting is the fact that only *CsLBD36_2* and *CsLBD42* were positively correlated with total catechins. Eight *CsLBDs* were found to have positive correlations with the other metabolites, while 19 had no correlations with the studied metabolites. These *CsLBDs* were distributed throughout all subclasses, except subclass Ib. *CsLBD6* and *CsLBD16_2* were negatively correlated with these metabolites.

Tea (*Camellia sinensis*) has a remarkable content, PAs are produced by the branched flavonoid pathway and can be classified as soluble or non-soluble. The relationship between *CsLBDs* and soluble PAs was opposite to that between *CsLBDs* and non-soluble PAs. Galloylated catechins (ECG, galloylated flavan-3-ols, EGCG cis-flavan-3-ols) and total catechins were correlated with *CsLBD* expression, and *CsLBD36_2* and *CsLBD42* expression were shown positive correlation with the accumulation of these metabolites. In addition, C, EC, *trans*-flavan-3-ols, GC, EGC and non-galloylated flavan-3-ols showed correlations with the expression of *CsLOB_3*, *CsLBD21*, *CsLBD11_2*, *CsLBD11_3*, *CsLBD12_3*. Detailed correlation analyses of *CsLBD* genes and metabolites is shown in Supplementary Dataset 3.

### Subcellular localization and transactivation activity analysis of CsLBDs

**Figure 7:**
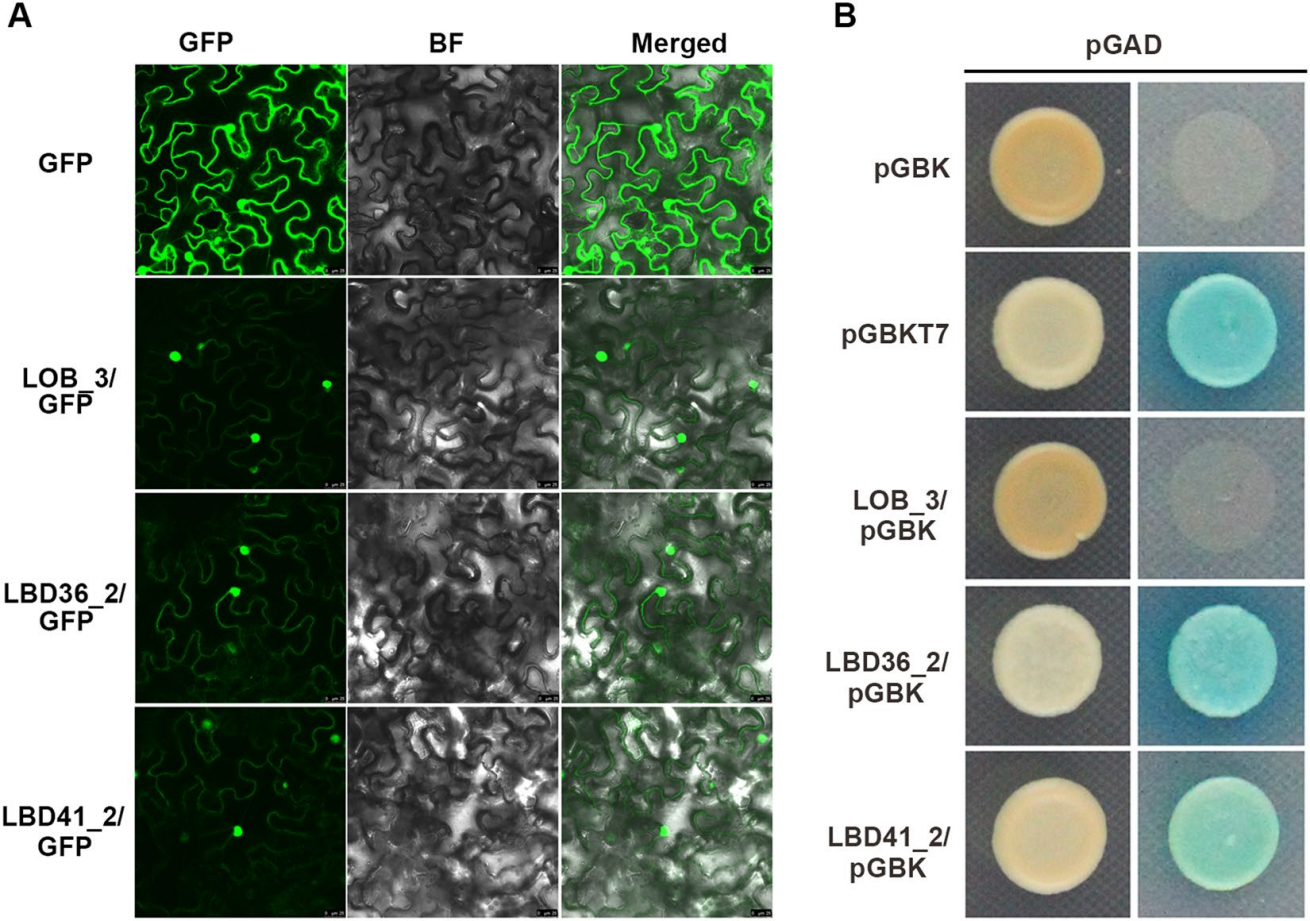
The potential function of LBDs in tea plant. **(A)** Subcellular localization of CsLOB_3, CsLBD36_2 and CsLBD41_2 and GFP as a control, which were transiently expressed in *N. tabacum* leaves. GFP: Green fluorescence image, BF: Bright-field microscopy image, Merge: Merged bright-field and green fluorescence images. **(B)** Transactivational analyses of CsLBDs in yeast. Positive control, negative control and the fusion constructs were transformed into the AH109 strain and successively incubated in SD/-Leu/-Trp media and SD/-Ade/-Leu/-Trp/-His plate supplemented with X-α-GAL.

Correlation analysis between the transcriptomic profile and metabolites suggested that *CsLOB_3* and *CsLBD36_2* are positively correlated with total PAs and catechines, respectively, whereas *CsLBD41_2* was negatively correlated with soluble PAs. *CsLOB_3* and *CsLBD36_2* were predominantly expressed in apical buds and young leaves, and *CsLBD41_2* accumulated in tea flowers and roots. We were particularly interested in the detailed function of LBD genes, and cloned these genes from the tea plant cultivar ‘Longjing 43’. The open reading frames (ORFs) were inserted into the GFP reporter gene under the control of the CaMV 35S promoter. The GFP recombinant constructs and the CsLOB_3-GFP, CsLBD36_2-GFP and CsLBD41_2-GFP fusion proteins were introduced into tobacco. CsLOB_3-GFP, CsLBD36_2-GFP and CsLBD41_2-GFP were specifically localized in the nucleus (Fig. 7A), which is consistent with the predicted role of these genes as TFs. The GFP signal from the empty vector showed ubiquitous distribution throughout the cell.

To examine the transactivation activity of CsLBDs, we made the constructs containing CsLOB_3, CsLBD36_2 or CsLBD41_2 together with a DNA binding domain and each CsLBD-pGBK-pGAD pair was individually co-transformed into yeast cells AH109. Co-transformation of pGBKT7 and pGAD was used as a positive control, while pGBK and pGAD were the negative control. All of these transformants could readily grow on the SD/-Leu/-Trp medium and the resulting colonies were further selected on quadruple dropout medium supplemented with X-α-Gal. As shown in Fig. 7B, CsLOB_3 and the negative control did not grow, whereas CsLBD36_2, CsLBD41_2 and the positive control grew well and turned blue. This indicated that only CsLBD36_2 and CsLBD41_2 had transcriptional activity in these yeast strains.

### Analysis of the promoter regions of the structural genes involved in the flavonoid pathway in tea plant

**Figure 8:**
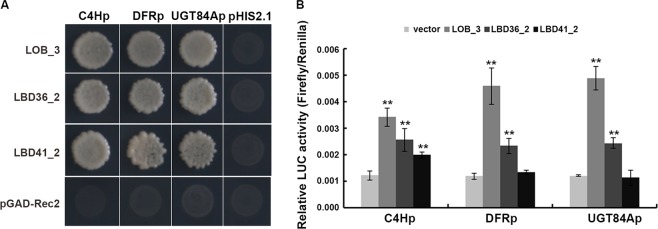
The transcriptional regulation of CsLBDs on the *CsC4H*, *CsDFR* and *CsUGT84A* promoter. **(A)** Yeast one-hybrid assays of the interactions between CsLBDs and *CsC4H*, *CsDFR* and *CsUGT84A* promoter fragments. The empty vectors of pGADT7-Rec were used as a negative control. The concentration of 3-amino-1,2,4-triazole was 30 mM. **(B)** Relative LUC/REN ratio from transient expression assays. The relative LUC activities were normalized to a 35S: REN internal control. Error bars indicate the SD of five biological replicates. **Significant difference at P < 0.01.

To explore the downstream molecular events behind the metabolic processes triggered by LBD TFs, we further analyzed the promoter regions of the structural genes involved in the flavonoid pathway. A total of 52 genes were analyzed and LBD dominant binding sites (G:HCGGCG or GCGGCW) were present in the promoter regions of three flavonoid biosynthesis related genes, TEA034002.1 (C4H), TEA010588.1 (DFR) and TEA026127.1 (UGT84A) (Supplementary Fig. S3). To identify whether CsLBD could bind to the promoter of these three structural genes, the *CsC4H*, *CsDFR* and *CsUGT84A* promoter regions were used as bait, and those yeast cells co-transformed with three CsLBD-pGADT7-Rec2 vectors, were tested on SD/-Trp/-Leu/-His + 30 mM 3-AT media, respectively (Fig. 8A). The result showed that all three CsLBDs bound to the *cis*-element in the promoter of *CsC4H*, *CsDFR* and *CsUGT84A*. We then cloned the promoter regions in a vector harboring the LUC reporter gene and analyzed the effect of CsLBDs on gene transcription. Compared to the background, the promoter activity of *CsC4H* was significantly elevated 2.8-fold, the *CsDFR* promoter was up-regulated by 3.8-fold and the *CsUGT84A* promoter was raised 4-fold when introduced to CsLOB_3. In addition, the LUC activity of three promoters was increased by different levels by CsLBD36_2, whereas CsLBD41_2 showed little impact on these promoter activities, only the *CsC4Hp* activity increased 1.6-fold (Fig. 8B). Taken together, our data suggest that CsLBD proteins are involved in flavonol biosynthesis.

## Discussion

Tea is the world’s most popular beverage and offers a wealth of health benefits^38,39^. Catechins, anthocyanidins and proanthocyanidins are important secondary metabolites that are synthesized via the flavonoid pathway^37^. In higher plants, flavonoid biosynthesis is not only regulated by structural genes but also involves a number of transcription factors. LBD transcription factors exist ubiquitously in plants and are involved in mediating plant-specific processes^14,42^. Following the completion of the tea plant genome sequence, 54 LBD genes from *Camellia sinensis* were identified. Comparative studies of other plant species show similar numbers of LBDs to that in *Camellia sinensis*, which indicates the evolutionary diversification of the LBD family through extensive expansion^43^.

Structural analysis is a powerful method of mining valuable information concerning protein function. The sequence alignment of 54 LBD proteins showed that all contained four Cys residues in the C block, except CsLBD29, which lacks the last three Cys residues. CsLBD23 and CsLBD24 lack the GAS-block, and the leucine zipper-like coiled-coil of CsLBD16_2, CsLBD2_1, CsLBD2_2, CsLBD7_1, CsLBD7_2, CsLBD26_1, CsLBD26_2, CsLBD27_1, CsLBD27_2 and CsLBD27_3 varied in amino acid content. Similar results were found in Soybean, *L. japonicus*^9,44^. However, LBDs in *Arabidopsis*, rice, apple and mulberry contain invariant residues in their conserved domains, indicating that LBD proteins in *Camellia sinensis* have more variation. Exon/intron structure and gene length analysis showed that most LBD genes in *Camellia sinensis* had similar gene structures within the same subgroup, a pattern also observed in *Arabidopsis*, rice and soybean^6,44^. We also demonstrated that CsLBD proteins generally possess similar protein motifs within each subgroup. Motif 1 contained the conserved CX2CX6CX3C zinc finger-like motif, motif 2 and motif 3 comprised the GAS-block and (D/N) PX2G motifs, respectively, and motif 4 contained the leucine zipper-like coiled-coil (LX6LX3LX6L) domain. Nearly all members of class I possessed motifs 1–4, whereas Class II contained motifs 1, 2, 3 and 5. This suggests that the CsLBDs in the same group or subgroup have similar functions to their homologs.

Recently, it has been reported that low temperature, NaCl, drought and other abiotic stressors promote the production of secondary metabolites in other species^45–48^. The identification of *cis*-elements showed that 31 *CsLBDs* have at least one of the HD-Zip1, G-box, GC-motif, MBS, MRE and WUN-motif elements, which are mainly involved in responses to abiotic stress and light. Eleven and seven *CsLBDs* were induced by NaCl and drought stress, respectively. The promoter of *CsLBD1_1*, *CsLBD1_2*, *CsLBD12_1*, *CsLBD13* and *CsLBD18* contained ABRE and MBS, which mainly participate in drought-inducibility, and all of them were accumulated after NaCl and drought treatments. Only six *CsLBDs* were downregulated after the low temperature treatment, suggesting that LBD may play diverse roles in response to different stresses. A total of 47 *CsLBD* promoters were found to have hormone responsiveness elements, including TATC-box and P-box elements related to gibberellin responsiveness, and ABRE and CGTCA elements, which are associated with abscisic acid and MeJA responsiveness, respectively. Recently, ABA has been reported to promote the biosynthesis of flavonols^49,50^. There were one to six ABRE elements distributed in the promoter of 27 *CsLBDs*, which indicates that these *CsLBDs* are regulated by ABA and flavonoid synthesis. It has also been reported that ABA and MeJA promote anthocyanin accumulation, also GA plays positive roles in the flavonoid pathway in apple and tea plant^51–53^. Expression analysis of *CsLBDs* after MeJA treatment, showed that only *CsLBD38* and *CsLBD39_2* were upregulated and ten *CsLBDs* were repressed by MeJA, five of which contained the gibberellin-responsive element in promoter region. Since expression pattern is closely related to the gene function^47,51^, these results suggested that these CsLBDs mainly repress flavonoid biosynthesis.

LBD proteins play central roles in a wide range of metabolic, physiological and developmental processes^5,7,17,25^. There may be a “bridge” between the transcription factor, the biosynthetic gene and the secondary metabolite, and integrated analyses of gene expression and metabolites to select key genes involved in metabolite processes have been reported^54–56^. We found that *CsLBD36_2* was highly correlated with total catechins, with a correlation coefficient of 0.79. *CsLOB_3* and *CsLBD21* showed clear positive correlations with soluble PAs, with correlation coefficients of 0.69 and 0.74, respectively, and 18 CsLBDs showed negative correlations with soluble PAs, with the correlation coefficient values were between −0.76 to −0.5. Combined promoter, stress response and correlation analysis of transcription and metabolites suggested that LBDs are involved in plant secondary metabolism, and act as hormones to mediate plant development and defense responses, which is consistent with other species^17,22^. There are extensive studies of the function of LBDs in plant growth and development, whereas knowledge about the mechanisms by which LBD genes control secondary metabolism is limited. Thus far, only *AtLBD37*, *AtLBD38* and *AtLBD39* are known to act as repressors of anthocyanin synthesis and N availability signals^7,21^. *OsLBD37* is also associated with nitrogen metabolism^7,24^. The functions of CsLOB_3, CsLBD36_2 and CsLBD41_2 in flavonoid synthesis were further researched, and were localized to the nucleus, while CsLBD36_2 and CsLBD 41_2 were shown to have self-activation activities. Previous studies showed that LBDs can dominantly recognize HCGGCG/GCGGCW to mediate the transcription of genes involved in plant growth, development and metabolic processes^7,54^. Here, we present that all three tested CsLBDs can directly bind to the *cis*-element in the promoter of *CsC4H*, *CsDFR* and *CsUGT84A* to regulate their transcriptomic level. Several class I members of the *AtLBD* family have been implicated in plant development, for example, *AtLOB* was shown to regulate early leaf development and *AtLBD36* functions in regulating proximaldistal patterning of petals^5,54^. Our results show that *CsLOB_3* and *CsLBD36_2* positively regulate flavonoid synthesis, suggesting that CsLBDs play distinct roles in *Camellia sinensis* flavonoid regulation.

## Conclusions

We identified and systematically analyzed 54 *CsLBDs* genes in the tea plant genome. Bioinformatics and expression pattern analysis of *CsLBDs* indicated that these genes have potential functions in growth, development and metabolic process in tea plant. Correlation analysis between the expression levels of *CsLBDs* and the content of secondary metabolites in the flavonoid pathway suggest that *CsLBDs* are involved in the flavonoid biosynthesis pathway. Moreover, candidate genes, *CsLOB_3* and *CsLBD36_2*, were confirmed to be involved in the flavonoid biosynthesis pathway. Our report provides an important foundation for further functional studies on the *CsLBDs* and contributes to illuminating the regulatory mechanisms of the flavonoid synthesis pathway in tea plant.

## Materials and Methods

### Characterization of the *CsLBD* gene family

Forty-three annotated *AtLBD* genes and proteins were downloaded from the *Arabidopsis* information resource (http://www.Arabidopsis.org/), and were used in a multiple database search against the tea plant genome, which was downloaded from the tea plant information archive (http://tpia.teaplant.org/index.html). The programs INTERPROSCAN, SMART, MOTIF and PLANTSP were employed to examine the protein sequences that were derived from the candidate *CsLBD* genes. The ExPASy proteomics server (http://expasy.org/) was used to predict the isoelectric points and molecular weights of the CsLBD proteins. The Clustal X1.83 program was employed to alignment the protein sequences of CsLBD, MEGA X was used to construct a phylogenetic tree by the neighbor-joining method with bootstrap set to 1000^57^. Gene structure display server2.0 was used to define the exon/intron structures of individual *CsLBD* genes by aligning the cDNA sequences to their corresponding genomic DNA sequences. MEME (http://meme-suite.org/) was used to characterize the conserved motifs of CsLBDs with the following parameters: the maximum number of motifs = 15.

### Analysis of promoter regions and *cis*-acting elements

Plant CARE (http://bioinformatics.psb.ugent.be/webtools/plantcare/html/) was used to determine the putative *cis*-acting element distribution in the 2000-bp promoter sequence of 54* CsLBDs* and 51 structural genes in the flavonoid pathway.

### Correlation of gene expression and metabolite accumulation analysis

Transcriptome data and metabolite data from tea cultivar Shuchazao were downloaded from the tea plant information archive (http://tpia.teaplant.org/index.html). Fragments per-kilobase of exon per million fragments (FPKM) was used to estimate the gene expression level in eight tea plant tissues (root, stem, old leaf, mature leaf, young leaf, apical bud, flower and fruit). The expression levels of CsLBD genes in each tissue were calculated using Log_10_(FPKM value). Mev4.9.0 (https://sourceforge.net/projects/mev-tm4/) was used to display the CsLBD expression patterns. The samples of eight tissues of tea cultivar Shuchazao in RNA-seq experiments were also used for detecting the metabolites by high-performance liquid chromatography (HPLC) analysis^39^. To screen LBDs associated with the main flavonoids, correlation analysis between *CsLBD* genes and 13 representative metabolites was performed using Pearson’s correlation coefficient. Correlations with the value of correlation coefficients |R| > 0.5 and a p-value < 0.05 were considered statistically significant.

### Expression analysis

For total RNA extraction from tea plants a Trizol reagent (Invitrogen) was used according to the manufacturer’s instructions. Purified RNA (1 μg) was reverse transcribed using the transcription system (Vazyme). Beacon Designer 7.0 was used to design primers for the *CsLBDs* of interest, the primers are listed in Table S1. Real-time PCR was performed on an ABI7900HT Sequence Detection System (Applied Biosystems, CA, USA) using SYBR Green (Roche, Switzerland) in accordance with the manufacturer’s instructions, and the gene expression level was normalized to Glyceraldehyde-3-phosphate dehydrogenase (GAPDH, accession number: KA295375.1).

### Subcellular localization of CsLBDs

Plant-mPloc (http://www.csbio.sjtu.edu.cn/bioinf/plant-multi/) was used to predict protein subcellular localization. The *CsLBD* sequences were amplified and cloned into the binary vector pCV-eGFP-N1 digested with *Kpn* I and *BamH* I (Supplementary Dataset 4). The recombinant binary constructs and pCV-eGFP-N1 (control) were individually introduced into *Agrobacterium tumefaciens* strain GV3101. Agrobacterium cultures carrying the recombinant vectors were grown overnight at 28 °C, cells were pelleted and resuspended in infiltration buffer (10 mM MgCl_2_, 10 mM MES and 150 µM acetosyringone) with OD600 = 1. The cell suspensions were incubated at room temperature and then infiltrated into 4–6 week old *N. benthaminan* leaves. Expression of fluorescent proteins was observed with a Leica TCS SP5 confocal laser scanning microscope system (Leica Microsystems, Bannockburn, IL, USA) at 48 h post-agroinfiltration. Fluorescence of GFP was observed at 495–545 nm.

### Analyses of CsLBD transactivation activity

The CsLOB_3, CsLBD36_2 and CsLBD41_2 sequences were cloned into the pGBKT7 vector and pGBKT7-53 + pGADT7-T acted as a positive control, while pGBKT7-Lam+ pGADT7-T was a negative control. The transactivation assay was performed following instructions given in the yeast transformation system 2 user manual. BD-CsLBDs and pGBKT7-53 were individually transformed into the yeast strain AH109 and selected on SD/-Leu/-Trp plates. Colonies were further transferred to selective SD/-Ade/-Leu/-Trp/-His/X-α-Gal medium and incubated at 30 °C for 3–5 days.

### Yeast one-hybrid assay

The PCR products of CsLOB_3, CsLBD36_2 and CsLBD41_2 were purified and inserted into a pGADT7-Rec vector, The truncated derivatives of the *CsC4H*, *CsDFR* and *CsUGT84A* promoters were inserted into a pHIS2.1 vector. CsLBD-pGADT7-Rec (pGADT7-Rec plasmid digested by *Sma* I) was co-transformed with bait vectors into yeast stain Y187 and plated on SD/-Leu/-Trp medium. Colonies were then transferred to SD/-Leu/-Trp/-His deficient medium with 30 mM 3-AT for 3 days.

### Dual-luciferase assay for CsLBDs

Dual-luciferase assays were performed as described previously^37^. The CsLBDs sequences were cloned into the YUKKS vector. The 1.5 kb promoter regions of *CsC4H*, *CsDFR* and *CsUGT84A* were cloned into a pGreenII 0800-LUC vector. 35S::REN (Renilla luciferase) in the vector was used as an internal control. The activities of the *CsC4H*, *CsDFR* and *CsUGT84A* promoters with and without the effector transcription factors were measured using a Dual-Luciferase Reporter Assay System (Promega) following the manufacturer’s instructions.

### Main conclusion

Our study presents a comprehensive characterization of the 54 CsLBDsin *Camellia sinensis* and demonstrates the involvement of CsLBDs in the regulation of flavonoid biosynthesis.

## Supplementary information

Supplementary information

Dataset 1

Dataset 2

Dataset 3

Dataset 4

## Data Availability

All supporting data can be found within the manuscript and its additional files.
