# Supplementary material for: Structural and functional insights into the LBD family involved in abiotic stress and flavonoid synthases in Camellia sinensis
Source: Sci Rep. 2019 Oct 30;9:15651. doi: 10.1038/s41598-019-52027-6 (PMC6821796; doi:10.1038/s41598-019-52027-6)
Supplement: Supplementary file 1 — Supplementary information [file 41598_2019_52027_MOESM1_ESM.pdf]

Structural and functional insights into the LBD family involved in abiotic stress and flavonoid synthases in *Camellia sinensis*

Xueying Zhang[1,#], Yuqing He[2,#], Wenda He[1], Hui Su[1], Yuefei Wang[1], Gaojie Hong[2,*] and Ping Xu[1,*]

1 Department of Tea Science, Zhejiang University, Hangzhou 310058, China

2 State Key Laboratory for Quality and Safety of Agro-products, Institute of Virology and Biotechnology, Zhejiang Academy of Agricultural Sciences, 198 Shiqiao Road 310021, China

[#] These authors contributed equally to this work

* Corresponding author Ping Xu (xuping2002@163.com) and Gaojie Hong (gjhong@126.com).

Supplementary Figures

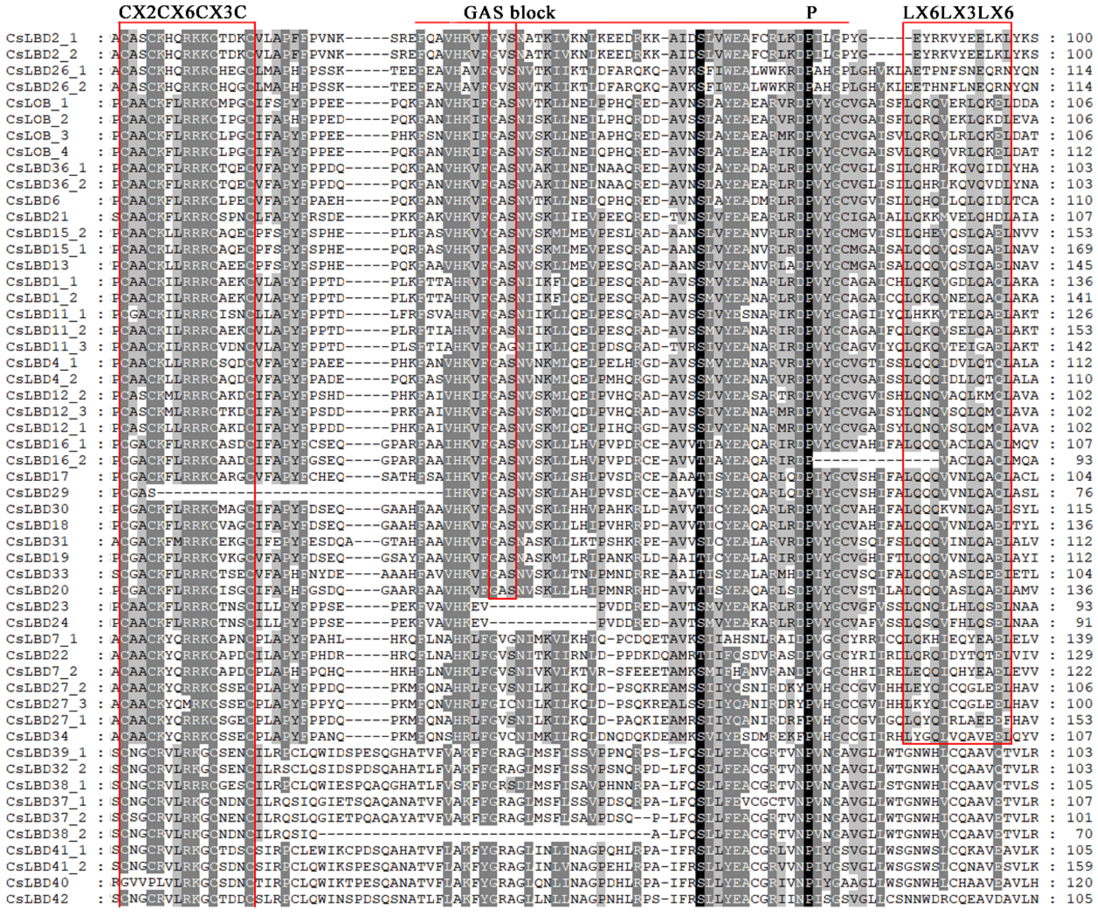

Supplementary Figure 1. Comparison of the amino acid sequences of CsLBDs.

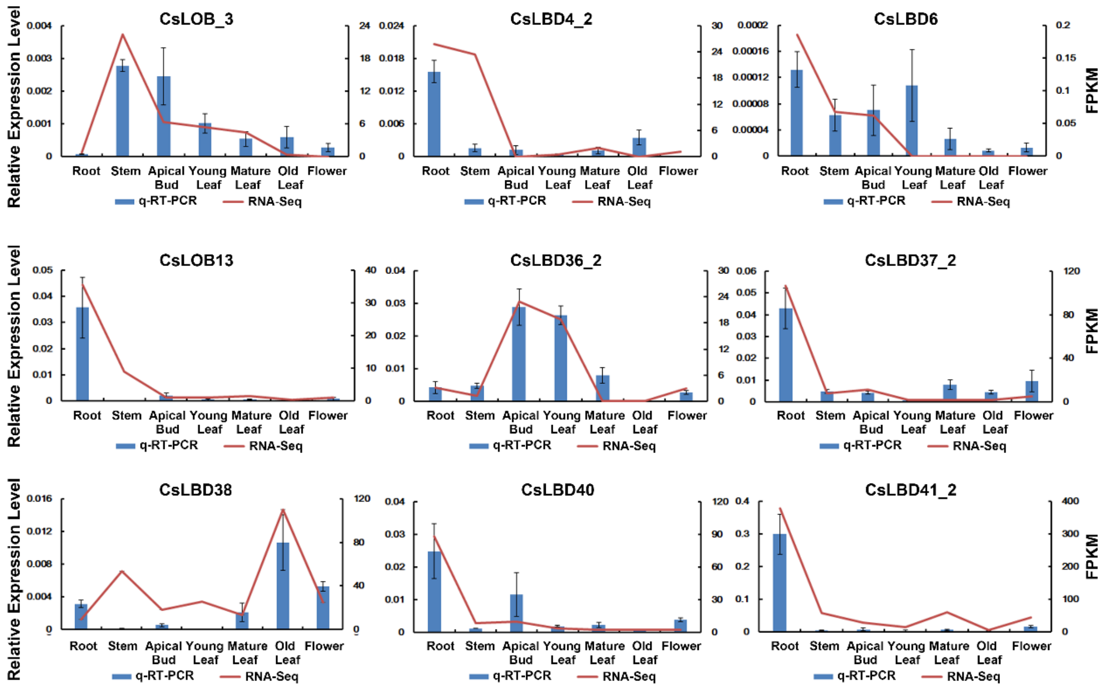

Supplement Figure 2. Expression patterns of the nine *CsLBDs* in different tissues.

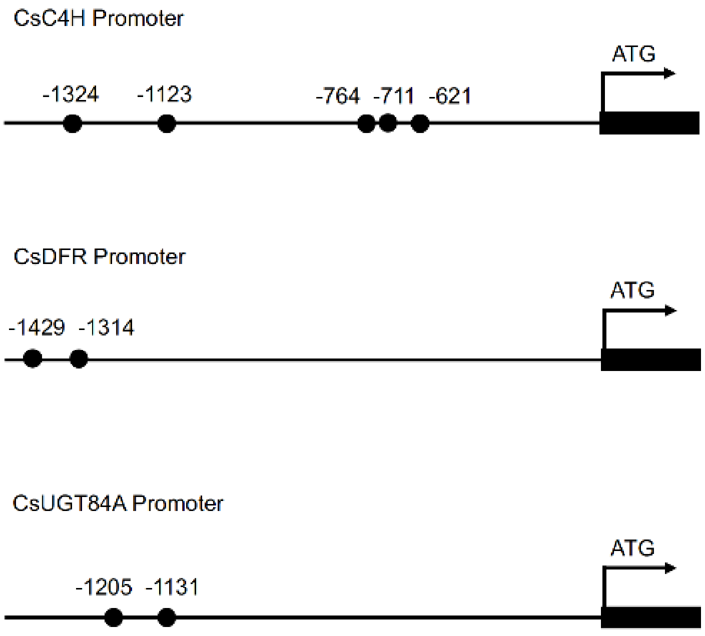

Supplementary Figure 3. Analysis of LBD binding site in the promoter region of structural genes involved in the flavonoid pathway.
